# Isolation and Characterization of Tissue-Derived Extracellular Vesicles from Mouse Lymph Nodes

**DOI:** 10.3390/ijms26136092

**Published:** 2025-06-25

**Authors:** Bernadett R. Bodnár, Sayam Ghosal, Brachyahu M. Kestecher, Panna Királyhidi, András Försönits, Nóra Fekete, Edina Bugyik, Zsolt I. Komlósi, Éva Pállinger, György Nagy, Edit I. Buzás, Xabier Osteikoetxea

**Affiliations:** 1 Institute of Genetics, Cell- and Immunobiology, Semmelweis University, 1089 Budapest, Hungary; bodnar.bernadett.95@gmail.com (B.R.B.); kiralyhidi.panna@gmail.com (P.K.); gyorgyngy@gmail.com (G.N.);; 2 HCEMM-SU Extracellular Vesicles Research Group, 1089 Budapest, Hungary; 3 HUN-REN-SU Translational Extracellular Vesicle Research Group, 1089 Budapest, Hungary; 4 Department of Rheumatology and Immunology, Semmelweis University, 1023 Budapest, Hungary

**Keywords:** extracellular vesicles, exosomes, lymph node, immunization, ovalbumin

## Abstract

Extracellular vesicles (EVs) are lipid membrane-enclosed particles released by all cells and can be isolated from various sources, even from solid tissues. This study focuses on isolating and characterizing EVs from mouse lymph nodes (LNs). Male C57BL/6 mice were injected with complete Freund’s adjuvant, with or without ovalbumin. Inguinal and popliteal LNs were incised 9 days after immunization, and EV isolation was carried out using a combination of differential centrifugation and size-exclusion chromatography. The characteristic morphology of small and large EVs was confirmed by transmission electron microscopy. Particle size distribution and concentration were determined by nanoparticle tracking analysis, while protein and lipid contents were measured by bicinchoninic acid assay, and sulfo-phospho-vanillin assays, respectively, to calculate the protein-to-lipid ratio. Immune and EV markers were analyzed by using flow cytometry and Western blot assay, revealing significant changes between immunized mice compared to controls. This study establishes a novel protocol for isolating and characterizing EVs from LNs and highlights the impact of immunization on EV properties, offering insights into their roles in immune processes.

## 1. Introduction

Extracellular vesicles (EVs) are a heterogeneous group of lipid bilayer-enclosed particles released by almost all cell types in physiological or pathological conditions.

Following the nomenclature recommended by the most recent MISEV 2023 guidelines, EVs can be classified based on their size as large (lEVs) or small (sEVs) EV subpopulations. EVs larger than 200 nm in diameter are considered lEVs, whereas those smaller than 200 nm are considered sEVs. A key characteristic of EVs is their diverse cargo, consisting of proteins, lipids, and nucleic acids that reflect their cellular origins [1,2]. Their ability to facilitate intercellular communication by transporting cargo from cell to cell has contributed to the rising interest in EVs in biomedical research, offering insights into various biological processes [1].

In addition, their roles in modulating immune responses have attracted great research interest, recognizing EVs as important players in various immune processes and as promising novel therapeutic modalities. Moreover, the immunological relevance of EVs originates from their participation in both pro-inflammatory and immunosuppressive processes [3]. These functions depend on their cellular origin and their conditions of release. EVs derived from different immune cells, such as dendritic cells and B cells, have been shown to play important roles in antigen presentation and immune activation [4,5,6,7]. Recently, studies have demonstrated that EVs can augment the antigen-presenting capacity by transferring antigens to resident antigen-presenting cells, therefore promoting adaptive immunity [8,9]. Such roles highlight EVs as important regulators of immune homeostasis and potential candidates for therapeutic application in immune disorders [10,11,12]. EVs can also serve as a platform for vaccine development, providing advantages of their delivery capability [13,14,15].

Ovalbumin (OVA) is a widely used model antigen for studying adaptive immune reactions in model organisms. Interestingly, engineered EVs with OVA have been shown to induce robust T-cell responses and generate long-lived memory T cells in murine models [16]. This is mediated by the trafficking of EVs with antigens to the draining lymph nodes (LNs). Complete Freund’s adjuvant (CFA) is a conventional adjuvant commonly used in adaptive immunization protocols in mice. This is due to CFA’s ability to activate antigen-presenting cells to efficiently induce T-cell responses [17,18].

LNs are crucial secondary immune organs, serving as the site of antigen presentation [19,20]. The presence of EVs in LNs suggests their important role in immune cell interactions, including intercellular communication between lymphoid and myeloid cells. These interactions could either amplify or suppress immune responses depending on the cellular origin, structure, and molecular cargo of EVs. However, to study EVs directly within the LNs, is substantially more challenging than separation of them from either conditioned media or body fluids. Here, the LN parenchyma needs to be effectively disrupted while preserving vesicle integrity and preventing the release of intracellular contents [21,22]. Standard protocols for tissue-derived EV separation recommend tissue homogenization and enzymatic digestion before isolating EVs using methods like differential centrifugation (DC), size exclusion chromatography (SEC), or density gradient ultracentrifugation (DGUC) [23,24,25,26]. Recent progress in tissue EV isolation has made EV characterization more reliable, enhancing our understanding of tissue-specific EV properties. Optimized enzymatic protocols have made it possible to separate different EV subpopulations from fibrotic tissue while preserving RNA and protein cargo in melanoma metastases [27]. Additionally, the validation of frozen tissue compatibility has enabled the high-purity isolation of EVs and confirmed the consistent size, morphology, and proteomic profiles between fresh and frozen samples. This approach enables the isolation of EVs even under laboratory conditions, where frozen samples are typically used, and fresh samples are not readily available [28]. Here, we separated EVs from inguinal lymph nodes (iLNs) and popliteal lymph nodes (pLNs) of mice after subcutaneous immunization given into the hind limbs.

Despite the progress in our understanding of the roles of EVs in vaccination and adaptive immunity, this topic is still relatively unexplored. Notably, there is very limited information on EVs inside LNs, where the most important steps of adaptive immunity are taking place. Therefore, our study is an important step forward to gain insight into the characteristics of EVs in LNs, and may contribute to the potential utilization of EVs in immunotherapy and vaccination.

## 2. Results

### 2.1. OVA + CFA Treatment Increases the Lymph Node Mass and Cell Number

**Figure 1:**
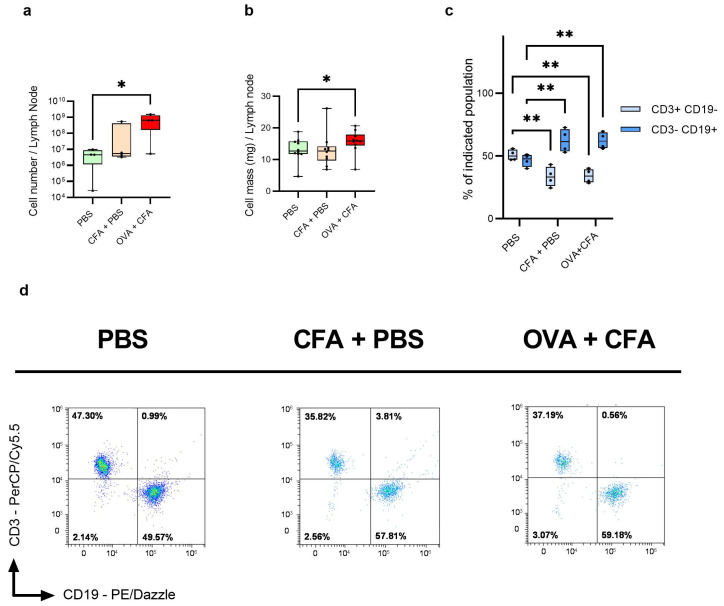
Quantifying the cell count (**a**) and mass (**b**) of isolated mouse inguinal lymph nodes (iLNs) and popliteal lymph nodes (pLNs). Viable LN cells were identified using an inverted microscope with trypan blue staining. Analysis of surface markers on isolated iLN and pLN cells by flow cytometry. The proportions of CD3^+^ CD19^−^ and CD3^−^ CD19^+^ surface-marked cells were quantified (**c**) the CD3^+^ CD19^−^ and the CD3^−^ CD19^+^ cells were gated similarly in each group and the gating was performed from the singlet population (**d**). Statistical significance of cell numbers and cell mass was determined by one-way ANOVA, * *p* < 0.05; n = 10 (cell mass), n = 4 (cell numbers). Statistical significance of surface markers of cells was calculated by two-way ANOVA, ** *p* < 0.001; n = 3.

On day 9 post-immunization, LNs were excised and OVA + CFA injection significantly increased LN mass (15.63 mg ± 3.71; *p* < 0.05) and cell numbers per mice (7.14 × 10^8^ ± 6.35 × 10^8^; *p* < 0.05) compared to the cell mass and cell number of the phosphate-buffered saline (PBS) control injected group. The CFA + PBS injected group did not show a statistically significant difference from the PBS-injected control group in total LN cell mass or total LN cell number (Figure 1a,b). Cell viability was above 85% across all groups, with no significant difference observed between the PBS-injected, CFA + PBS and OVA + CFA-injected groups (Supplementary Figure S1a,b).

### 2.2. Immunization Reduces the CD3^+^ CD19^−^ T Cell Number and Increases the Number of CD3^−^ CD19^+^ B Cells as Compared to Controls

Immunophenotyping of LN cells demonstrated that the proportion of CD3^+^ CD19^−^ T cells (34.05% ± 5.61 vs. 50.65% ± 4.26; *p* < 0.01) and CD3^−^ CD19^+^ B cells (62.25% ± 6.54 vs. 46.57% ± 4.95; *p* < 0.01) significantly changed following the OVA + CFA immunization as compared to the PBS-injected controls. Notably, the CFA + PBS injection resulted in a significant decrease in the CD3^+^ CD19^−^ T cell population (33.51% ± 7.95; *p* < 0.01) and an increase in the CD3^−^ CD19^+^ B cell ratio (62.08% ± 9.37; *p* < 0.01) as compared to the PBS controls (Figure 1c,d). The proportion of CD4^+^ CD8^−^ helper T cells and CD4^−^ CD8^+^ cytotoxic T cell ratios did not show significant difference across PBS, CFA + PBS and OVA + CFA-treated groups (Supplementary Figure S2).

### 2.3. Transmission Electron Microscopy (TEM) Revealed lEV and sEV Morphology

**Figure 2:**
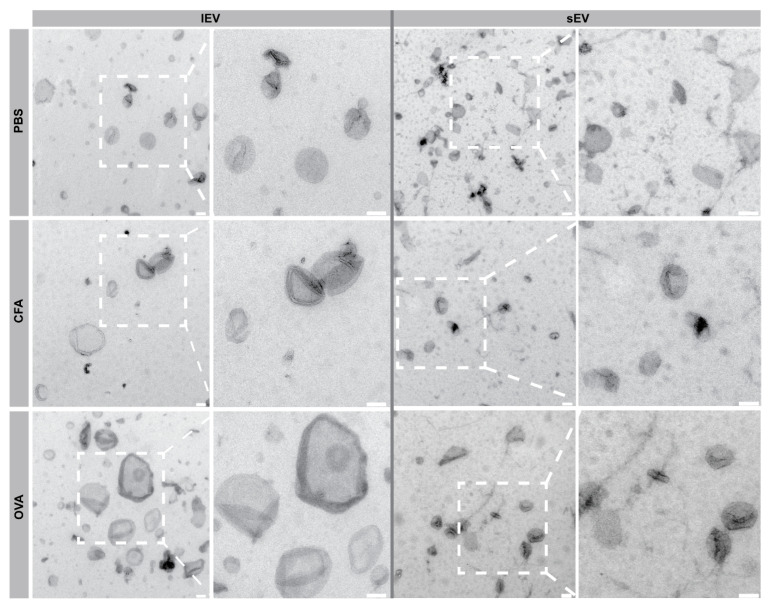
The morphology and heterogeneity of large extracellular vesicles (lEVs) and small extracellular vesicles (sEVs) following differential centrifugation (DC) and size exclusion chromatography (SEC) purification after phosphate-buffered saline (PBS), complete Freund’s adjuvant + phosphate-buffered saline (CFA + PBS), and ovalbumin + complete Freund’s adjuvant (OVA + CFA) immunization, as assessed by TEM. Dotted boxes highlight zoomed panels. Scale bars represent 100 nm.

TEM was used to demonstrate the feasibility of isolating intact EVs from the lymph nodes of mice and to characterize the morphology of the isolated EVs (Figure 2). To determine which isolation method provides less tissue-derived contaminants, EVs were analyzed using TEM after DC isolation alone, as well as after combining DC with SEC, which revealed that the SEC step is required to decrease tissue-derived materials such as fibers (Supplementary Figure S3). After DC and SEC isolation, the lEVs exhibited a heterogeneous population with a predominant size larger than 200 nm, featuring lipid bilayer-enclosed structures with typical EV morphology. Similarly, the sEVs, were predominantly smaller, below 200 nm with a distinct cup shape morphology and well-preserved membrane integrity (Figure 2).

### 2.4. sEVs Show Higher Particle Counts than lEVs with Variable Trends Across Groups

**Figure 3:**
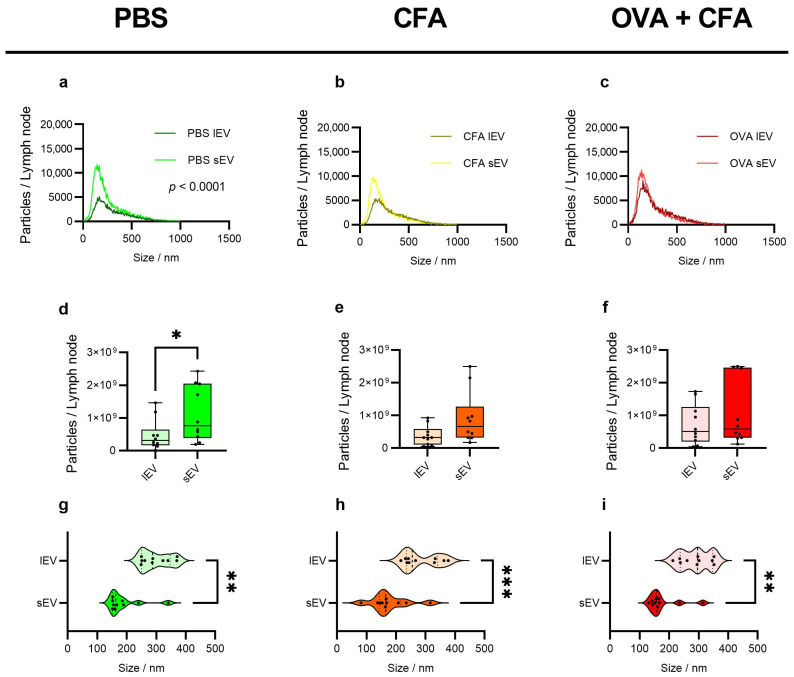
Size distribution of the lEV and sEV subpopulation measured by nanoparticle tracking analysis (NTA). The size distribution for the PBS control- (**a**), CFA + PBS- (**b**), and the OVA + CFA-treated groups (**c**). Boxplot representing the particles/lymph node subpopulations of EV assessed by NTA. Graphs depicting lEVs and sEVs of PBS control (**d**), CFA + PBS group (**e**), and OVA + CFA-treated group (**f**). The median size of the particles for the PBS control- (**g**), CFA + PBS- (**h**), and the OVA + CFA-treated groups (**i**). Statistical significance calculated by paired *t*-test, * *p* < 0.05; ** *p* < 0.01; *** *p* < 0.001; n = 10.

The size distribution analysis revealed that sEVs had increased counts per lymph node compared to lEVs isolated from PBS-treated animals (lEVs: 1.25 × 10^3^ ± 1.30 × 10^3^ vs. sEVs: 2.40 × 10^3^ ± 3.05 × 10^3^; *p* < 0.0001) (Figure 3a), but CFA + PBS or OVA + CFA injected animals did not show increased counts of sEVs compared to lEVs (Figure 3b,c). Analysis of the total particle numbers per LN from mice showed that the PBS group had significantly higher sEVs (1.1 × 10^9^ ± 8.5 × 10^8^) compared to the lEVs (4.7 × 10^8^ ± 4.7 × 10^8^; *p* < 0.05) (Figure 3d). A non-significant trend was observed, with CFA + PBS sEV particles showing higher counts than CFA + PBS lEVs (Figure 3e). Similarly, animals injected with OVA + CFA demonstrated the highest particle counts of sEVs compared to lEVs (Figure 3f). Median, mode, and mean central tendency differed among all groups and subpopulations. The median particle size was significantly larger for lEVs in all groups (PBS: 299.00 size/nm ± 48.67, *p* < 0.01; CFA + PBS: 284.20 size/nm ± 60.17, *p* < 0.001; OVA + CFA: 289.10 size/nm ± 51.10; *p* < 0.01) compared to sEVs in all groups (Figure 3g–i). The same was observed for the mode size of sEVs (PBS: 163.70 size/nm ± 58.73; *p* < 0.01; CFA + PBS: 162.00 size/nm ± 64.94; *p* < 0.001), which were smaller than lEV particles, while the OVA + CFA group did not differ in the sEVs and lEVs (Supplementary Figure S4a–c). The mean particle size of sEVs (PBS: 222.40 size/nm ± 67.93; *p* < 0.001 vs. CFA + PBS: (207.00 size/nm ± 75.9; *p* < 0.0001 vs. OVA + CFA: 205.00 size/nm ± 67.39; *p* < 0.01) was also lower than lEVs in all the three groups (Supplementary Figure S4d–f).

### 2.5. sEVs Show Higher Protein Content and Protein-to-Lipid Ratio than lEVs Across Groups, with Minimal Difference in Lipid Content

**Figure 4:**
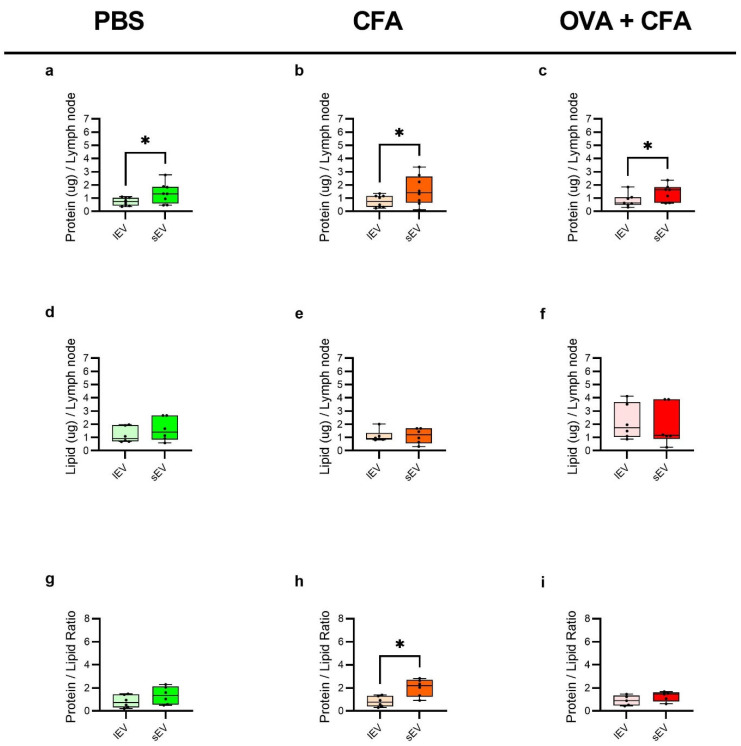
Boxplots demonstrating total protein content measured by BCA assay method, total lipid content measured by SPV assay and the ratio of protein per lipid content. The protein content in lEV and sEV subpopulations derived from the PBS control group (**a**), the CFA + PBS group (**b**), and the OVA + CFA-treated group (**c**). total lipid content measured by SPV assay. Distribution of total lipid content among lEV and sEV subpopulations of PBS- (**d**), CFA + PBS- (**e**), and OVA + CFA group (**f**). Furthermore, the ratio of total protein content to total lipid content in the PBS- (**g**), CFA + PBS- (**h**), and OVA + CFA (**i**) groups. Statistical significance was calculated by paired t-test, * *p* < 0.05; n = 8 (total protein content), n = 6 (total lipid content and protein content per lipid content).

The protein content per LN exhibited significant changes between lEVs and sEVs in each treatment group. In the PBS-injected group, sEVs showed significantly higher protein content compared to lEVs (1.38 µg/LN ± 0.77 vs. 0.73 µg/LN ± 0.29; *p* < 0.05) (Figure 4a). Similarly, in the CFA + PBS-injected group, sEVs displayed significantly increased protein levels relative to lEVs (1.59 µg/LN ± 1.11 vs. 0.77 µg/LN ±0.45; *p* < 0.05) (Figure 4b). Lastly, in the OVA + CFA-treated group, a significant difference was also observed in protein content between sEV and lEV (1.42 µg/LN ± 0.64 vs. 0.84 µg/LN ± 0.51, *p* < 0.05) samples (Figure 4c). In contrast, the total lipid content per LN did not differ significantly between lEV and sEV fractions across all experimental groups (Figure 4d–f).

In all three experimental groups, sEVs demonstrated enrichment in protein-to-lipid ratios. No change in the protein-to-lipid ratio was observed in the PBS group (Figure 4g), and OVA + CFA group (Figure 4i). In contrast, the CFA + PBS-injected group exhibited a significant change in protein-to-lipid ratio (lEVs: 0.81 ± 0.46; sEVs: 2.01 ± 0.75; *p* < 0.05) (Figure 4h). The analysis of protein per particle revealed consistent values across the experimental groups with no significant difference between lEVs and sEVs in PBS, CFA + PBS, or OVA + CFA group (Supplementary Figure S5a–c). Similarly, analysis of lipid per particle of lEV and sEV samples in the PBS and the OVA + CFA-treated group did not differ except for the CFA + PBS group, where lEVs (3.9 × 10^−3^ pg ± 2.0 × 10^−3^; sEVs: 1.5 × 10^−3^ pg ± 1.1 × 10^−3^; *p* < 0.05) had significantly elevated lipid per particle values compared to sEVs (Supplementary Figure S5d–f). Additionally, EV yields were also quantified as particles per mg of LN which revealed statistically significantly more particles per mg of LN in sEVs compared to lEVs for PBS and CFA + PBS groups (Supplementary Figure S5g–i).

### 2.6. Surface Marker Profiling Reveals Distinct Immunological Signatures on lEVs and sEVs with Significant Shifts in CD146, MHC II, and CD45 upon OVA + CFA Treatment

**Figure 5:**
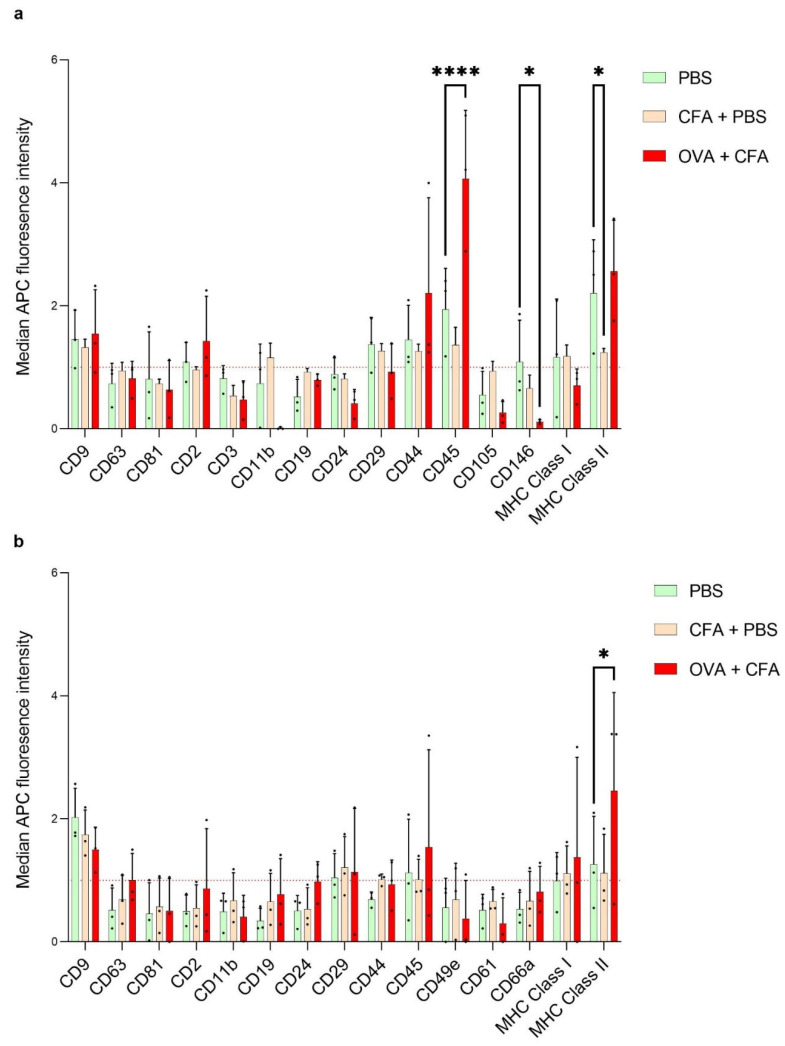
MACSPlex APC bead-based EV kit for immuno-oncology for detection of potential surface markers of EV subtypes. The mean fluorescence intensity of allophycocyanin (APC) markers was evaluated and normalized relative to the average of conventional extracellular vesicle markers (CD9, CD63, CD81). Samples above fold change 1 could be relative quantity in the three measured groups. Graphs illustrate the 12 most abundant surface marker levels in lEV (**a**) and sEV subpopulations (**b**), where they are above a fold change of 1. Graph Statistical analysis of significance was calculated by two-way ANOVA, * *p* < 0.05; **** *p* < 0.0001; n = 3.

To assess the presence of various markers on our LN EVs, we next used bead-based surface marker analysis for our LN EVs. In lEVs, a significant decrease was observed in the CD146 surface marker expression (MFI: 1.09 ± 0.67 vs. 0.11 ±0.03; *p* < 0.05) between the PBS and OVA + CFA groups. Similarly, a significant increase was detected in the MHC Class II expression (MFI: 2.20 ± 0.87 vs. 1.24 ± 0.06; *p* < 0.05) between the PBS and CFA + PBS groups. Furthermore, a highly significant increase was perceived in CD45 between the PBS and OVA + CFA-treated mice (MFI: 1.94 ± 0.66 vs. 4.06 ± 1.11; *p* < 0.0001) (Figure 5a). In the case of sEVs, MHC Class II was significantly increased in OVA + CFA compared to the PBS group (MFI: 2.45 ± 1.59 vs. 1.26 ± 0.78; *p* < 0.05). Additionally, we show the expression levels of the most abundant immune surface markers in both subpopulations for the OVA + CFA groups (Supplementary Figure S6a,d). In addition to the above markers, some were less abundant in lEVs but showed statistically significant differences between PBS and OVA + CFA groups, including CD4 (MFI: 0.57 ± 0.25 vs. 0.26 ± 0.27; *p* < 0.05), CD8a (MFI: 0.62 ± 0.13 vs. 0.15 ± 0.11; *p* < 0.001), CD61 (MFI: 0.40 ± 0.23 vs. 0.06 ± 0.05; *p* < 0.05), and CD326 (MFI: 0.43 ± 0.31 vs. 0.07 ± 0.01; *p* < 0.01) (Supplementary Figure S7a). However, in sEVs, we did not detect any further significant differences in the less abundant markers between the PBS and OVA + CFA groups (Supplementary Figure S7b). Finally, Western blot analysis was performed to confirm the presence of extracellular vesicles by detecting characteristic EV-associated protein markers. We validated the presence of typical EV markers in both l- and sEVs, including CD9, CD63 and CD81 as tetraspanin membrane protein markers, as well as Alix and TSG-101 cytosolic proteins involved in vesicle biogenesis. We also confirmed the absence of the calnexin as a negative endoplasmic reticulum marker (Supplementary Figure S8).

## 3. Discussion

In this study, we characterized EVs isolated from iLNs and pLNs from solid tissue after the immunization of mice. Immunization was performed with PBS alone as a control group, CFA + PBS as an adjuvant control group, and OVA + CFA as a-treated group. In addition, we combined DC and SEC isolation methods to achieve higher EV purity. Using various EV characterization methods, we examined the unique properties of lEVs and sEVs as distinct size-based subpopulations.

An important aspect was the selection of an appropriate method to generate the immune response in LNs. Immunization with OVA emulsified in CFA is a widely used method to study immune responses in mice. This approach not only induces a robust immune reaction but also elicits significant alterations in the draining LNs. Following OVA + CFA immunization, there is a noticeable increase in the size and cellularity of LNs. For instance, in wild-type mice, LN cell counts increased approximately fourfold from baseline within two days post-immunization in previous reports [29,30]. Our experimental results are consistent with these previous findings, as we observed a significant increase in both the cell mass and cell count of draining LNs following OVA + CFA immunization, compared to the control group that did not receive the immunogen and adjuvant emulsion (Figure 1a,b). Furthermore, immunization with OVA + CFA also influences the cellular composition of the LNs. Following immunization, the draining LNs undergo changes, including an increase in B cell and plasma cell interactions, which are important for inducing an effective antibody response during infections [31]. In our experiments, immunophenotyping revealed a significant increase in the CD3^−^ CD19^+^ B cell and a decrease in CD3^+^ CD19^−^ T-cell populations in both CFA + PBS-treated and OVA + CFA-treated mice (Figure 1c).

EVs are essential regulators of intercellular communication and have significant roles in physiological and pathological processes. Isolation of EVs from solid tissues ex vivo is especially challenging; however, it presents a unique opportunity to study their biological roles [23,32]. It is important to process tissues under conditions that closely resemble the in situ environment and to minimize their exposure to altered conditions.

LNs are hubs of immune activity, and the EVs released from them may carry important cargo contributing to adaptive immunity. These characteristics make lymph node-derived EVs exceptional for studying immune responses while preserving EV functionality. Unlike plasma- or serum-derived EVs, LN-derived EVs are less likely to be ‘diluted’ by EVs secreted from much more abundant cell types, such as platelets and endothelial cells, or by other factors present in those biofluids.

In our study, a key consideration was minimizing sample loss and achieving a higher yield due to the small size and mass of the mouse LNs. This consideration also prevented the use of additional downstream isolation methods such as DGUC or tangential flow filtration. Instead, our choice of DC and SEC as the downstream isolation method has been previously demonstrated to be suitable in another study of EVs from human LNs and mouse spleens [24].

Using TEM imaging, we provided evidence for the feasibility of EV isolation from murine LNs and characterized their morphology. Our TEM analysis revealed that the EV counts were reduced using the additional SEC isolation compared to DC alone, but significantly the amount of tissue-derived material observed in EV samples (Supplementary Figure S3). The choice of isolation method should depend on the intended application of the EV preparations. For example, in some applications, yield may need to be prioritized over purity, and UC alone may suffice. However, in many applications, it is crucial to avoid contamination by non-EV constituents such as protein or lipid complexes, which may interfere with proteomic analysis or functional assays. Our aim was to isolate and characterize ex vivo EVs from mouse LNs. For this reason, we favored the purity achieved by SEC over differential centrifugation alone (Supplementary Figure S3).

In addition to establishing an appropriate method for isolating EVs from LN solid tissue, our aim was to characterize and compare the morphology of the purified lEV and sEV subpopulations in response to immunization (Figure 2). Throughout the characterization, we strictly followed the MISEV2023 guidelines of the EV research [1]. In our study, we identified the fundamental differences between lEVs and sEVs following immunization, including their morphology, concentration, size distribution, protein and lipid composition, and their relative ratio, as well as their vesicle surface markers. In PBS control mice, we detected a significantly higher number of sEVs per lymph node. However, this difference was not observed in either the CFA + PBS or the OVA + CFA-immunized groups. This suggests that immunization results in a diversification of EV subpopulations, leading to increased heterogeneity (Figure 3d–f).

Among the vesicle surface markers assessed, we found that CD45, CD146 and MHC Class II were differentially expressed on EVs. This is especially interesting considering the immunological roles of CD45 in T cell receptor signaling [33] and of MHC Class II in antigen presentation [34]. Previous research has demonstrated that murine intestinal epithelial cells respond to ovalbumin-induced immune stimulation by functionally altering their exosomal characteristics—specifically, by upregulating MHC class II molecules and directing exosomes toward gut-associated lymphoid tissues [35]. Notably, MHC Class II transfer by EVs has been reported by others in cell cultures but not in LN tissue [36,37,38,39,40]. Our experimental results support a similar conclusion, showing significant molecular alterations post-immunization. Notably, we also observed upregulation of CD45 in lEVs, which reflects a regulatory mechanism potentially involved in optimizing T cell activation and memory formation [41]. Moreover, an increased surface density of MHC class II molecules was detected on sEVs. This raises the question of whether EVs isolated from draining lymph nodes after immunization play a functional role in active antigen processing and presentation.

It should be noted that our study had some challenges. Among other aspects, the isolation of LNs from PBS-injected mice requires substantial expertise, particularly in properly excising the iLNs and pLNs without the surrounding connective tissue. Therefore, in our experiments, the pLNs from the PBS control group were not excised and were not included in our measurements. Following immunization, the LNs became swollen, particularly in the OVA + CFA-treated group. When measuring cell surface markers, we focused on four lymphoid markers, excluding myeloid markers due to the major predominance of lymphoid cell populations in the LNs. However, myeloid markers of much lower abundance may be of interest for future studies that include affinity enrichment.

While there is much promise in studying EVs directly from tissue, isolating them presents unique challenges due to biological heterogeneity, technical limitations, and methodological variability compared to more established sources such as cell media supernatants or blood. An important challenge is the relatively lower yield of tissue-derived EVs. Furthermore, mechanical procedures required for tissue processing, such as homogenization can rupture cells, releasing intracellular vesicles that contaminate EV preparations. While gentle dissociation methods help preserve EV integrity, they often reduce yield or may fail to adequately dissociate the tissue. In our experiments, the small size of mouse lymph nodes made manual mechanical disaggregation particularly difficult. This step is more feasible in larger organs. Additionally, enzymatic treatments used during tissue dissociation, such as collagenase or DNase, can degrade EV surface markers like CD81. This can alter the proteomic profile of the isolated EVs, complicating downstream characterization and reducing the reliability of biomarker studies [32]. Altogether, rather than relying on a general protocol for EV isolation from tissue, it is essential to develop and apply protocols that are tailored to the specific tissue type. Additional avenues of future research can also be in the study of the cargo of different subpopulations, the analysis of their nucleic acid profiles, or examining potential changes in the cytokine profiles secreted by the cells.

## 4. Materials and Methods

### 4.1. Mice

All mouse experiments were conducted according to the institutional guidelines of the Council Directive of the European Union (86/609/EEC). Ethics approval was obtained from Semmelweis University’s Institutional Animal Care and Use Committee (PEI/001/1781-3/2015) under the guidelines outlined by the National Food Chain Safety Office (Act CLXXXVIII of 2012) for the care and use of laboratory animals, which were firmly followed. Male wild-type mice (C57BL/6J, RRID: IMSR_JAX:000664, The Jackson Laboratory) aged 6–20 weeks were used for every experiment. The breeding, housing, and care took place in a conventional animal facility with a temperature and humidity-controlled environment, 12-h light–dark cycle and mice had *ad libitum* access to GLP-certified mouse feed (VRF-1, Special Diets Services) in the Institute of Genetics, Cell- and Immunobiology, Semmelweis University, Hungary.

### 4.2. Immunization of the Mice

The animals were randomly selected into three groups including a PBS-injected control group (n = 7), CFA + PBS-injected controls (n = 5), and an OVA + CFA treatment group (n = 5). Due to the limited amount of lymph node tissue for each group, we pooled lymph nodes from seven mice for control PBS groups or five mice per group for the CFA + PBS and OVA + CFA groups. For immunization of the OVA + CFA-treated group, a stable emulsion was prepared on the day of immunization by emulsifying 1 mg/mL CFA (Imject™ Freund’s complete adjuvant (FCA), 77140, Thermo Fisher Scientific, Waltham, MA, USA) with PBS containing 1 mg/mL OVA (10 mg per formulation, OVA EndoFit, vac-pova, InvivoGen, San Diego, CA, USA) in a 1:1 ratio [42]. To ensure adequate immune response and consistent drainage to the iLNs and pLNs, 50 µL immunogenic adjuvant emulsion was administrated subcutaneously into the skin of the dorsal foot area for each hind limb of mice with an insulin syringe (1 mL, U40 insulin, 29G × ½”, 0.33 × 12.7 mm, Chirana, Stará Turá, Slovakia).

### 4.3. LN Isolation and Cell Extraction

On day 9 post-immunization, the PBS, the CFA + PBS and the OVA + CFA injected group of mice were euthanized by carbon dioxide inhalation. In the case of mice injected with PBS alone, only the iLNs were incised (Supplementary Figure S9). In comparison to the PBS group, both iLNs and pLNs were efficiently excised from mice immunized with CFA + PBS or OVA + CFA, LNs were pooled for each group in 5 mL Eppendorf^™^ tubes filled with 1 mL 1× PBS placed on the ice during the whole procedure. Next, the organs were weighed and stored in cold 0.1 µm filtered 1× PBS on ice for further steps. The experiment was conducted following the protocol described earlier [25], without any modifications. Concisely, the regional LNs were mechanically minced into approximately 2 mm × 2 mm × 2 mm tissue cubes under sterile conditions. To extract tissue vesicular structures, the previously minced tissue fragments were digested with a mixture of Collagenase D (Collagenase D, 100 mg per formulation, 11088858001, Merck, Rahway, NJ, USA, 2 mg/mL) and DNAse I (DNase I, 100 mg per formulation, 11284932001, Merck, Rahway, NJ, USA, 40 U/mL) by gentle agitation on a shaker (Hybaid Midi Oven Dual 14 Oven with Shaker) with 24 rpm at 37 °C for 30 min. After the incubation, the tissue pieces in the medium were transferred through a 70 µm sterile cell strainer (EASYSTRAINER 70 µM, FOR 50 ML TUBES, 542070, Greiner Bio-One, Kremsmünster, Austria) allowing the liquid to drain by gravity. The collected liquid was then centrifuged at 300× *g* for 10 min at 4 °C (Eppendorf^®^ Centrifuge 5804R, 5805000010, Hamburg, Germany). The pellet was analyzed for cell immunophenotyping and the supernatant was filtered through a 5 µm sterile filter (Millex—SV 5.00 µm, 0000289930, Sigma Aldrich, St. Louis, MO, USA) to exclude any remaining cells and for further investigation.

### 4.4. Immunophenotyping of LN Cells

The 300 g pellet was washed once in 0.1 µm filtered 1× PBS, resuspended in 1 mL 0.1 µm filtered 1× PBS and the cells were counted after trypan blue (trypan blue solution, 20 mL, T8154-20ML, Merck, Rahway, NJ, USA) staining using a Bürker chamber. Cells (1 × 10^6^) were transferred into the new tube and the non-specific Fc receptors blocking was performed using CD16/CD32 Fc-block antibody (purified anti-mouse CD16/32, 50 µg per formulation, 1106505, Sony Biotechnology, San Jose, CA, USA) in accordance with recommended protocol by the manufacturer (Supplementary Table S1). Following the incubation period, cold 2% FBS (Fetal Bovine Serum, F9665-50ML, Sigma Aldrich, St. Louis, MO, USA) in 0.1 µm filtered 1× PBS was added followed by 450× *g* centrifugation for 5 min at 4 °C. The pellet was stained with the appropriate concentration of antibodies based on the manufacturer’s recommendations (Supplementary Table S1). The stained cells were incubated in the dark for 30 min at 4 °C. Following the incubation, the cells were washed and cold 0.1 µm filtered 1× PBS was added to a final volume of 100 µL. Cell viability was assessed using a viability marker (eBioscience™ Fixable Viability Dye eFluor™ 780, 100 tests per formulation, 65-0865-14, Thermo Fisher Scientific, Waltham, MA, USA) following the manufacturer’s protocol (Supplementary Figure S1a,b). The measurement was conducted using a flow cytometer (CytoFLEX S N2-V3-B5-R3 Flow Cytometer, B78557 Beckman Coulter, Inc., Brea, CA, USA). For calibration, we used a calibration bead (CytoFLEX Ready to Use Daily QC Fluorospheres, 2 × 10 mL C65719, Beckman Coulter, Inc., Brea, CA, USA). To generate the compensation matrix required for the measurement, we used the VersaComp antibody capture bead (VersaComp Antibody Capture Kit, 2 Vials, LUO, B22804, Beckman Coulter, Inc., Brea, CA, USA). Gating optimization buffer control and unstained control samples were measured additionally. The results were analyzed using CytExpert software (version 2.4.0.28, Beckman Coulter, Inc., Brea, CA, USA).

### 4.5. LN EVs Isolation by Differential Centrifugation Method

Isolation of EVs by DC followed the previously described protocol [25] with a modification. In brief, the supernatants were obtained from 300× *g* for 10 min at 4 °C centrifugation and then applied filtration with a 5 µm sterile filter (Millex™-SV Filter Unit (Sterile), SLSV025LS, Merck, Rahway, NJ, USA) by gravity on ice. After, the supernatants were repeatedly centrifuged at 2000× *g* for 20 min at 4 °C (Eppendorf^®^ Centrifuge 5425 R, Eppendorf Group, Hamburg, Germany). From the previous centrifugation, the pellets were discarded, and the supernatants were filtered through a 0.8 µm syringe filters (Whatman^®^ Puradisc 30 syringe filters, 50/pk, WHA10462240, Sigma Aldrich, St. Louis, MO, USA) by gravity on ice. Additionally, the filtrated supernatants were centrifugated with 16,500× *g* for 20 min at 4 °C (Hermle Z326 K Refrigerated Centrifuge, Hermle AG, Gosheim, Germany), and the obtained lEV pellets were resuspended into 100 µL 0.1 µm filtered 1× PBS. The supernatants were further processed with ultracentrifugation at 118,000× *g* for 2.5 h at 4 °C (Optima XPN-100 Ultracentrifuge, B10048, Beckman Coulter, Inc., Brea, CA, USA), and the extracted sEV pellets were resuspended in 100 µL of 1× PBS for subsequent SEC purification.

### 4.6. LN EVs Purification by SEC

70 nm isolation range qEV single columns (qEV single Columns, 20 packs per box, ICS-70, IZON, Christchurch, New Zealand) were used as SEC-based commercially available columns. The collection of fractions was performed according to the optimal protocol described by the manufacturer, with modifications implemented by us. Initial experimental observations indicated that EV-containing fractions were collected at an earlier stage (Supplementary Figure S10). Hence, we collected 0.530 mL default buffer volume as Void and continued with 0.850 mL fractions containing purified EVs. In brief, a series of 0.2 µm filtrated buffers as 1× PBS, 1× ultrapure water (Elix^®^ Essential Water Purification System, Merck, Rahway, NJ, USA) with 20% EtOH, and 0.5M NaOH were freshly filtrated through 0.2 µm syringe filter (Fisherbrand™ Sterile PES Syringe Filter, 50/pk, 15206869, Fisher Scientific, Waltham, MA, USA). The columns were washed three times with 3 mL of 1× PBS to ensure the properly washed condition of the columns. Then, the entire 100 µL resuspended pellets from 16,500× *g* centrifugation (lEVs) and the 100 µL resuspended pellets (sEVs) from 118,000× *g* ultracentrifugation were layered separately into each membrane of the columns dedicated for the different groups. After the sample had penetrated the membranes 1× PBS was added and the 0.530 mL void volume was collected followed by the 0.850 mL lEV and sEV fractions. The remaining fractions contained high-protein peaks that were collected in separate tubes according to their respective fractions. After fractions were harvested and 1× PBS reached the membrane, the column was washed with 3 mL of 0.5 M NaOH followed by 3 mL of 1× PBS. The column was stored with 1 X ultrapure water (Elix^®^ Essential Water Purification System, Merck, Rahway, NJ, USA) and 20% EtOH mixture at 4 °C. The collected 0.850 mL fractions of lEVs and sEVs were subjected to repeated centrifugation for concentration using parameters appropriate for the respective subpopulations. The 0.850 mL fraction containing lEVs was centrifuged at 16,500× *g* for 20 min at 4 °C, while the fraction containing sEVs was concentrated by ultracentrifugation at 118,000× *g* for 2.5 h at 4 °C. The pellets obtained after centrifugation were resuspended in 50 µL of 0.1 µm filtered 1× PBS.

### 4.7. TEM Analysis of LN EVs

For TEM imaging, we applied the previously described EV isolation method with minor modifications [43]. Both previously centrifugated concentrated lEVsand sEV pellets were resuspended in 0.1 filtrated 10 µL 1× PBS instead of 50 µL final volume. After, isolated EV samples were fixed in 2% paraformaldehyde (Paraformaldehyde, powder, 95%, 158127, Sigma Aldrich, St. Louis, MO, USA) in 1× PBS for 30 min at 4 °C. Then, they were incubated for 15 min on glow-discharged at 7.2 V for 60 s, carbon-coated 100-mesh copper grids using a Bal-Tec MED 020 Coating System. After washing with 1× PBS and fixating with glutaraldehyde 1% (Sigma-Aldrich, St. Louis, MO, USA) for 5 min at 4 °C, grids were washed again and dried with filter paper. The grids were treated with 2% aqueous uranyl acetate (Sigma-Aldrich, St. Louis, MO, USA) for 2 min to generate negative staining. Samples were washed and dried, then analyzed with FEI Tecnai G2 Spirit TEM (Thermo Fisher Scientific) equipped with a Morada digital camera (Olympus Soft Image Solutions GmbH, Muenster, Germany). All TEM procedures were undertaken at the University of Valencia’s Electron Microscopy facility.

### 4.8. Particle Size Distribution Analysis

The size distribution, as well as the mean, mode, and median central tendency, alongside particle concentration for both lEVs and sEVs, were quantitatively characterized through nanoparticle tracking analysis (NTA). All measurements were performed under the following settings: the temperature was 25 °C with a laser wavelength of 520 nm and conductivity of 15,000 uS/cm, 11 positions (2 cycles per position). The instrument operated in these properties: auto exposure, sensitivity 60, shutter speed: 100, minimum brightness 20, maximum brightness: 255, minimum area: 5, maximum area 1000. Properties of the camera: FpSec 30, number of cycles: 2, FRate 7.50000, gain: 24.00000 for lEVs and 28.80000 for sEVs, offset: 0. The analysis was conducted using the ZetaView (S/N: 19-459 ZetaView Particle Tracking Analyzer PMX-120 instrument, Munich, Germany, employing the ZetaView Analysis 8.05.12 SP2 software).

$$\frac{Concentration\;\left(\frac{Particle}{mL}\right)\;\times \;Volume\;(mL)}{Number\;of\;Lymph\;nodes\;(n)}$$

### 4.9. Total Protein Content Quantification

The protein concentration of EV subpopulations was determined with a micro BCA protein assay kit (Micro BCA™ Protein Assay Kit, 500 mL per kit, 23235, Thermo Fisher Scientific, Waltham, MA, USA) with some minor modifications. In brief, each of the standards, buffer control, and 10 X diluted EV samples were mixed with Micro BCA working solutions provided by the manufacturer and 0.5% Triton X-100 (TRITON™ X-100, (Polyethylene glycol tert-octylphenyl ether), 1L, M143-1L, VWR International, Radnor, PA, USA) and were worked on ice, and incubated at 60 °C for 1 h. The absorbance measurement was conducted with a spectrophotometer (NanoDrop 1000 Spectrophotometer with NanoDrop 1000 Version 3.8.1 software, Thermo Fisher Scientific, Waltham, MA, USA). The standard curve was created using absorbance values of the standards and the protein concentration of each unknown EV sample was calculated following the guidelines provided the instructions by the manufacturer.

$$\frac{Concentration\;\left(\frac{mg}{mL}\right)\times Volume\;(mL)}{Number\;of\;Lymph\;nodes\;(n)}$$

### 4.10. Total Lipid Content Quantification

Total lipid analysis was performed using the sulfo-phospho-vanillin (SPV) assay on ice in Eppendorf tubes and transferred into a non-treated 96-well microplate (Nunc™ MicroWell™ 96-Well Microplates, 108 per box, 243656, Thermo Fisher Scientific, Waltham, MA, USA) for incubation. The experiments were conducted as described earlier [44,45]. In brief, 1 µg/uL DOPC (1,2-dioleoyl-sn-glycero-3-phosphocholine, lyophilized powder, P6354-25MG, Sigma Aldrich, St. Louis, MO, USA) liposome was employed for a standard calibration. Serial dilutions were prepared in eight 1.5 mL Eppendorf tubes, with a volume of 40 µL in each tube. The listed volumes of DOPC were taken into the tubes: 0; 0.25; 0.5; 1; 2; 4; 8, and 16 µL, and the final volume was augmented with freshly filtered 0.1 µm 1× PBS buffer. From the isolated subpopulations, 10 µL of EV samples diluted in 20 µL with 1× PBS were pipetted into Eppendorf tubes. For safety, 100 µL of 96% sulfuric acid (339741, Sigma Aldrich, St. Louis, MO, USA) was added to each tube under a chemical hood. After transfer into a non-treated 96 well microplate (96 well plate, non-treated surface, pack of 25, 266120, Thermo Fisher Scientific, Waltham, MA, USA), the incubation was performed for 20 min at 90 °C with 350–400 rpm shaking. After the incubation time elapsed, the samples were placed at 4 °C for 5 min. Working towards under the chemical hood, phospho-vanillin reagent was prepared by dissolving 50 mg vanillin (ReagentPlus^®^, 99%, 79617, Merck, Rahway, NJ, USA) in 50 mL of 17% phosphoric acid (85 wt. % in H_2_O, 99.99% trace metals basis, 345245-100ML, Merck, Rahway, NJ, USA). 60 µL of phospho-vanillin was added to each well containing the standard and the examined samples. Then, a shaking step was applied at 350–400 rpm with a temperature of 37 °C for 1 h, catalyzing the color reaction. In the last step, after the second incubation period, we determined the absorbance at 540 nm using an ELISA reader (352 Multiskan MS LabSystems Microplate Reader, 74165-5, Labsystem, Vantaa, Finland). A standard curve was calibrated using the absorbance values of the measured standards. The lipid concentration of each unknown sample was determined and calculated with the help of the standard curve.

$$\frac{Concentration\;\left(\frac{ug}{uL}\right)\times Volume\;(µL)}{Number\;of\;Lymph\;nodes\;(n)}$$

### 4.11. Calculation of the Protein per Particle, Lipid per Particle, Protein to Lipid Ratio, and EV Yield

To determine the protein per lipid ratio, the previously measured total protein content was divided by particle number, expressed as LN equivalents. The lipid per particle ratio was calculated as the total lipid content divided by the total particle number represented per LN. The protein-to-lipid ratio was calculated as follows: previously described total protein values were divided by the calculated total lipid ratio per LN of the three different subgroups. The EV yield was determined by dividing the total particle number by the mass (mg) of lymph nodes.

### 4.12. Bead-Based Analysis of LN EVs

The MACSPlex EV Kit (MACSPlex EV Kit IO, mouse, up to 20 tests, 130-122-211, Miltenyi Biotech, Miltenyi Biotec Bergisch Gladbach, Germany) was used to detect 37 different surface epitopes for all of the isolated lEV and sEV subpopulations, including two isotype controls. total protein concentration was calculated by micro BCA assay (Micro BCA™ Protein Assay Kit, 500 mL per kit, 23235, Thermo Fisher Scientific, Waltham, MA, USA). Then, 15 µL total protein concentrated samples were used for each reaction. The experiment followed the overnight protocol provided by the manufacturer. Measurements were performed using CytoFLEX (CytoFLEX S N2-V3-B5-R3 Flow Cytometer, B78557 Beckman Coulter, Inc., Brea, CA, USA). Measurement was taken at 30 µL/min speed. The gating strategy was established based on our previously published study [46]. All the samples were analyzed by subtracting the negative bead control from each detected surface marker event. After averages were calculated of tetraspanin markers (CD9, CD63, CD81) then normalized the signal intensities for each EV marker for comparison of the profiles by adapting the different signal ranges.

### 4.13. Western Blot Analysis

We applied Western blot analysis to confirm the presence of EV-related marker proteins. For effective membrane protein extraction, vesicles were lysed using 0.1% Triton X-100 (TRITON™ X-100, (Polyethylene glycol tert-octylphenyl ether), 1L, M143-1L, VWR International, Radnor, PA, USA) in PBS and incubated on ice for 10 min. Following lysis, total protein content was measured using a micro BCA assay (Micro BCA™ Protein Assay Kit, 500 mL per kit, 23235, Thermo Fisher Scientific, Waltham, MA, USA). Approximately 10 µg of protein was mixed with 4× Laemmli buffer (4× Laemmli Sample Buffer, 1610747, Bio-Rad, Hercules, CA, USA) supplemented with 10% β-mercaptoethanol (2-Mercaptoethanol, M6250, 10 mL, Merck, Rahway, NJ, USA) to create a reducing environment. For CD63 detection, samples were run under non-reducing conditions, excluding β-mercaptoethanol. Samples were incubated at 100 °C for 5 min and immediately placed on ice for 5 min. After treatment, 15 µL of each sample was loaded per well onto polyacrylamide gel (4–20% Mini-PROTEAN^®^ TGX™ Precast Protein Gels, 15-well, 15 µL, 4561096, Bio-Rad, Hercules, CA, USA). A protein ladder (Precision Plus Protein Dual Color Standards, 500 µL, 1610374, Bio-Rad, Hercules, CA, USA) was used as a molecular weight marker. Following electrophoresis, proteins were transferred to a PVDF membrane (Immun-Blot PVDF Membrane, Roll, 26 cm × 3.3 m, 1620177, Bio-Rad, Hercules, CA, USA). After transfer, the PVDF membrane was pre-treated with an enhancer solution (SuperSignal™ Western Blot Enhancer, 46640, Thermo Fisher Scientific, Waltham, MA, USA). Membrane blocking was performed using 5% skimmed milk powder dissolved in washing buffer for 1 h. After washing with washing buffer, primary antibodies were added at the appropriate concentrations and incubated overnight at 4 °C according to the listed parameters (Supplementary Table S1). The next day, membranes were washed again with washing buffer and incubated with HRP-conjugated secondary antibodies, which were diluted in washing buffer containing 1% skimmed milk powder at appropriate concentrations (Supplementary Table S1). Following 1 h of incubation at room temperature, chemiluminescent signals were detected using an ECL Western blotting substrate (Clarity™ Western ECL Substrate, 500 mL, 1705061, Bio-Rad, Hercules, CA, USA) and visualized with ChemiDoc imaging system (ChemiDoc XRS+ System, Bio-Rad, Hercules, CA, USA).

### 4.14. Software and Statistical Analysis

Data ware handled with Microsoft Excel (Microsoft Office Professional Plus 2016 (16.0.5474.1000) and statistical analysis was conducted using GraphPad Prism for Windows (Version 9.0.0). Values are displayed as mean ± standard deviation, and data were considered significant when *p* values were <0.05. A normality test was used to determine whether the sample data were drawn from a normally distributed population in all groups. For all groups exhibiting normal distribution, a paired t-test was performed, and for those groups that were not exhibiting normal distribution, the Wilcoxon matched-pairs signed rank test was applied. For comparison of the potential interactions between two data sets, a two-way analysis of variance (ANOVA) was performed with Šídák’s multiple comparisons test to determine significant differences within groups. Schematic figures were made using Biorender https://www.biorender.com/ (accessed on 2 April 2025) and Microsoft PowerPoint (Microsoft Office Professional Plus 2016 (16.0.4266.1001)).

## 5. Conclusions

The iLNs and pLNs isolated from OVA + CFA-immunized mice were subjected to immunophenotyping, followed by successful isolation of the lEV and sEV subpopulations. TEM confirmed the feasibility of isolating intact EVs from murine LNs and characterized their morphology. While SEC reduced EV concentration compared to differential centrifugation, it significantly improved sample purity by reducing the extent of contamination. Based on these findings, combining DC for initial EV concentration with SEC for final purification is recommended to optimize yield and purity for downstream applications. Subsequently, downstream analyses were performed, where we examined the morphology, concentration, size, protein and lipid composition, as well as molecular markers. Our results suggest that immunization alters the composition of LN-derived EVs, and the characteristic profiles of the lEV and sEV populations often differ. Our results suggest that markers such as CD45 and CD146 on lEVs, as well as MHC Class II on sEVs are differentially abundant after immunization. Our current work offers an opportunity to better understand the characteristics of EV subpopulations within the draining LNs of mice and support the further development of human protocols and translational studies as well for immunotherapy and vaccine development.

## Data Availability

Data is contained within the article and supplementary materials.

## Supplementary Materials

The following supporting information can be downloaded at: https://www.mdpi.com/article/10.3390/ijms26136092/s1.
