# Supplementary material for: Isolation and Characterization of Tissue-Derived Extracellular Vesicles from Mouse Lymph Nodes
Source: Int J Mol Sci. 2025 Jun 25;26(13):6092. doi: 10.3390/ijms26136092 (PMC12249715; doi:10.3390/ijms26136092)
Supplement: Supplementary file 1 [file ijms-26-06092-s001.zip › ijms-3620598-supplementary.pdf]

Supplementary figures:

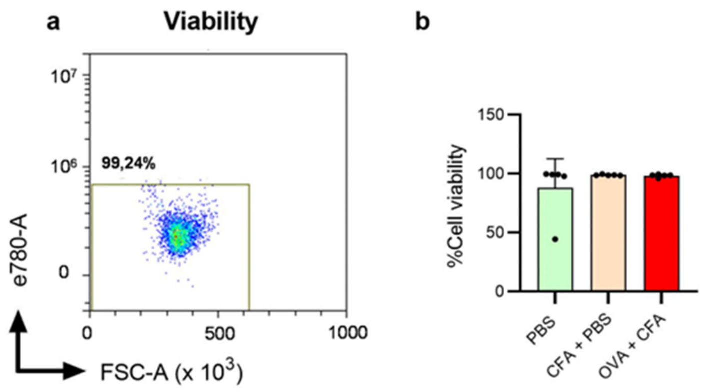

Supplementary Figure S1: Quantified the viable cell ratio from the isolated LNs cells measured by flow cytometry using viability dye. The dot plot of living cells was gated from the cell population (**a**) and the bar graph presents of viability each isolated groups (**b**). Graph Statistical analysis was calculated by one-way ANOVA; n = 5.

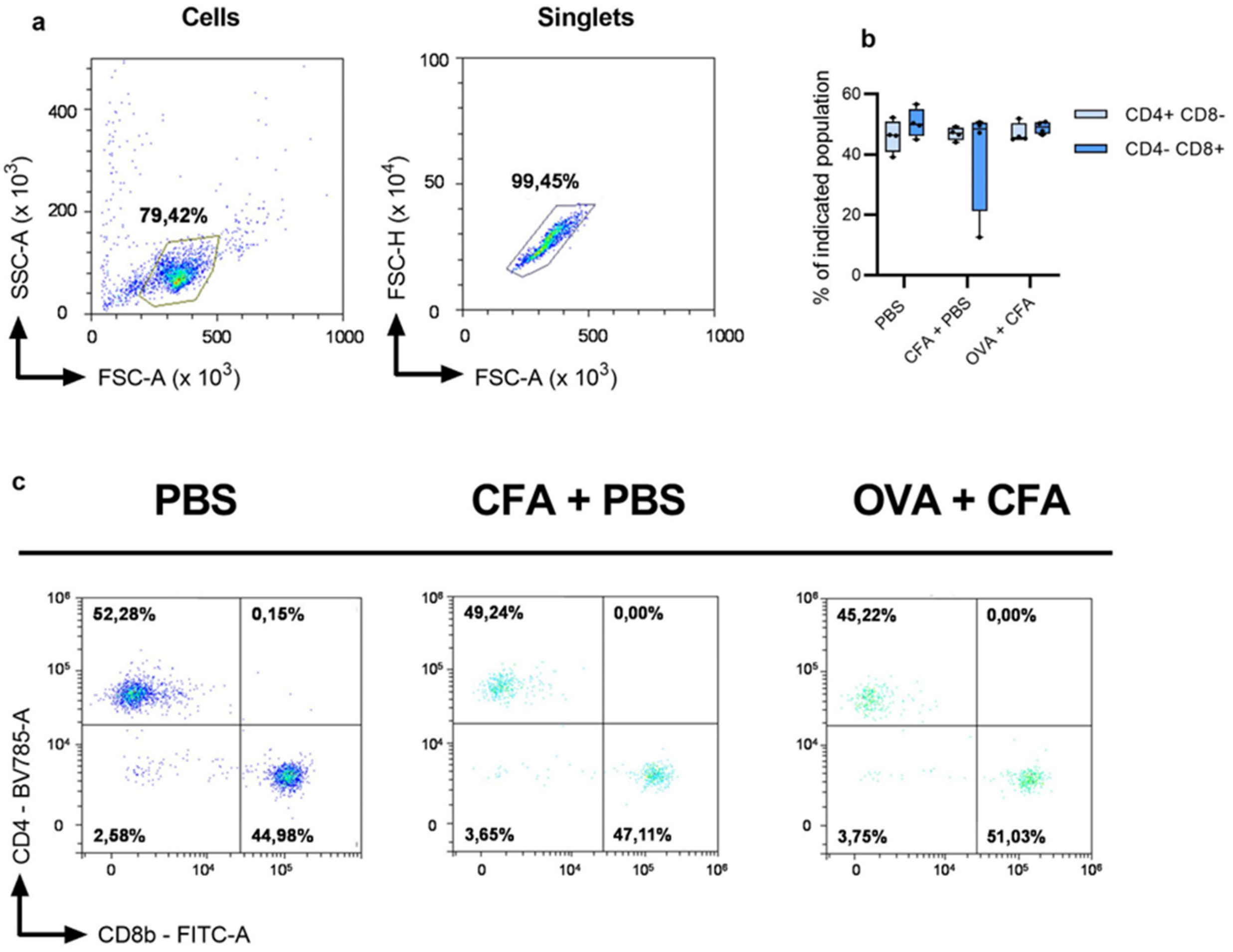

Supplementary Figure S2: Gating strategy of surface markers on isolated inguinal and popliteal LN cells by flow cytometry. Central gating strategy of cell populations and singlets represented as a pseudo-color plot used to identify the populations, applied in all measurements (**a**). Proportions of $CD4^+$ $CD8^-$ and the $CD4^+$ $CD8^-$ surface markered cells were identified from $CD3^+CD19^-$ and quantified (**b**). The gating strategy for identifying $CD4^+CD8^-$ helper T cells and $CD4^-CD8^+$ cytotoxic T cells in each group (**c**). Statistics were performed using two-way ANOVA; n = 3.

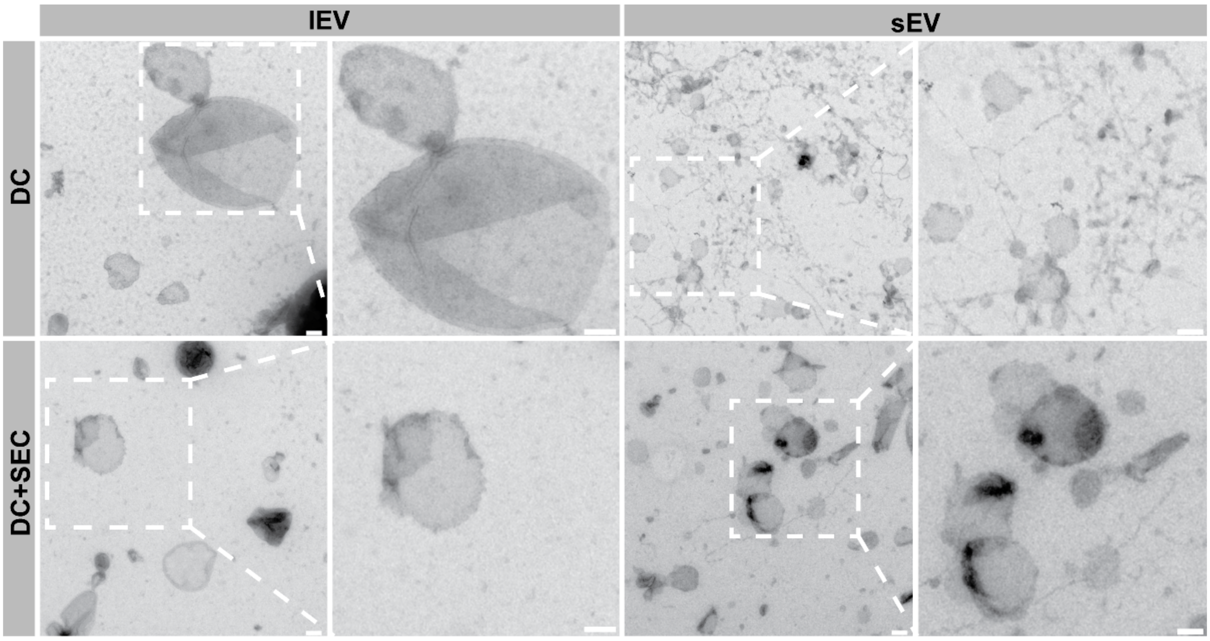

Supplementary Figure S3: TEM display of extracellular vesicle morphology and heterogeneity following isolation by differential centrifugation and differential centrifugation combined with size-exclusion chromatography. Scale bars represent 100 nm.

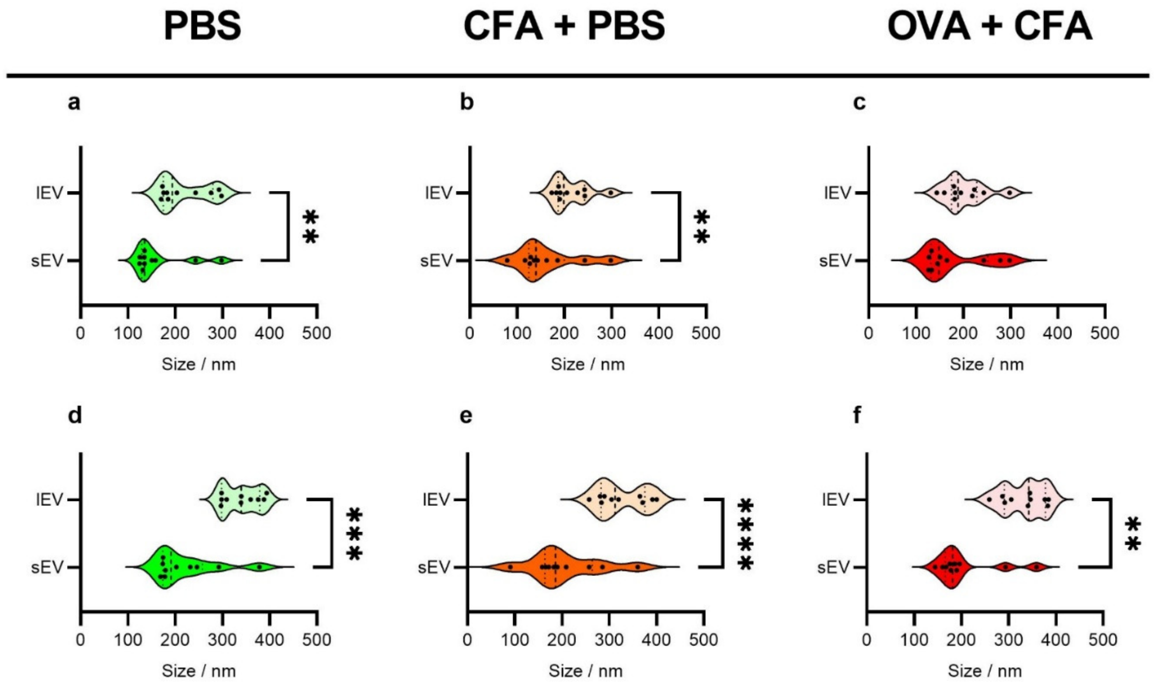

Supplementary Figure S4: The measure of central tendency of the lEV and sEV subpopulations analyzed by Nanoparticle Tracking Analysis. The mode size for the PBS control- (**a**), CFA + PBS- (**b**), and the OVA + CFA treated groups (**c**). The mean size for the PBS control- (**d**), CFA + PBS- (**e**), and the OVA + CFA treated groups (**f**). Statistical significance calculated by paired t-test, ** $p < 0.01$; *** $p < 0.001$; **** $p < 0.0001$; n = 10.

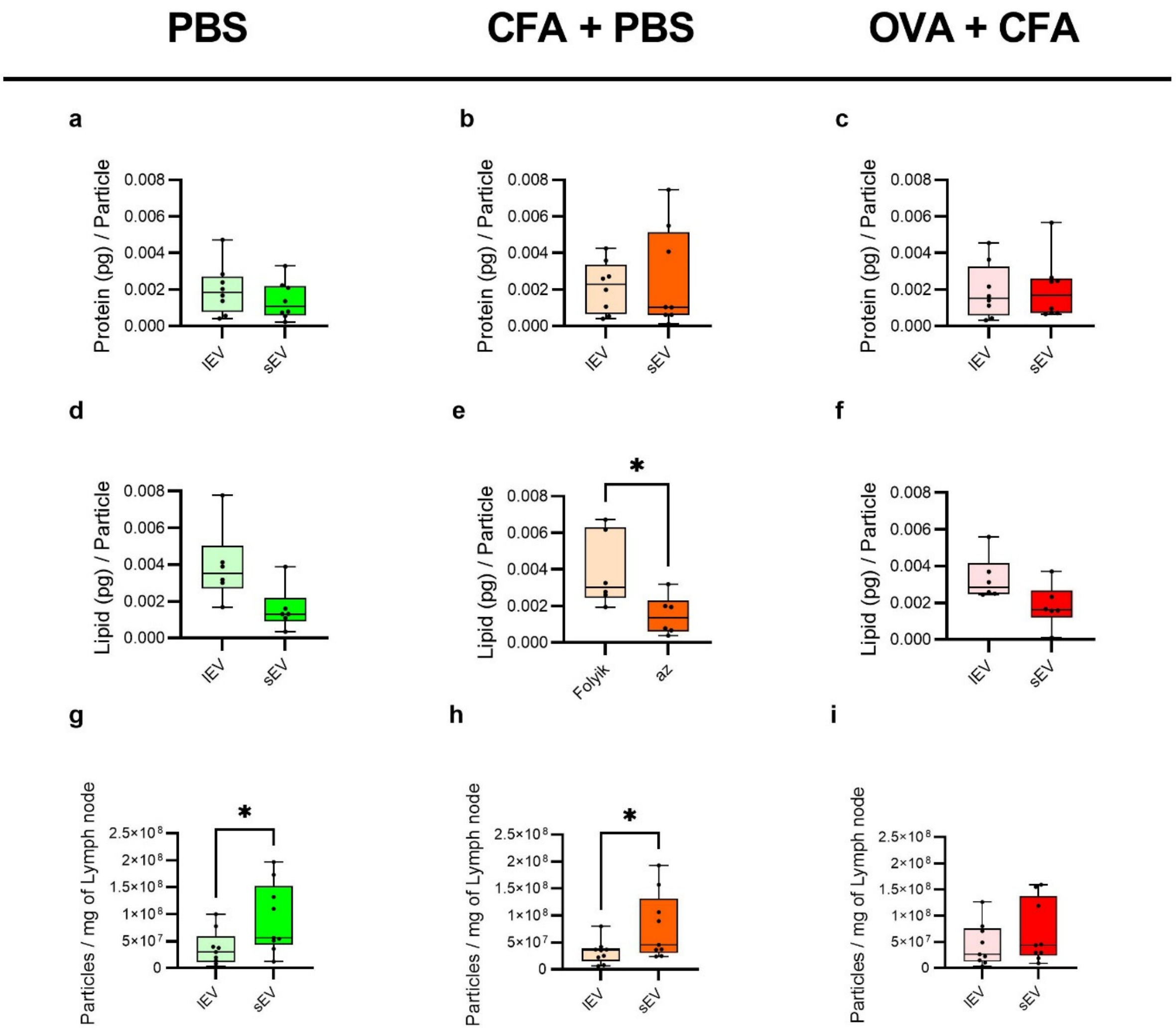

Supplementary Figure S5: The ratio of protein per particle, lipid per particle, and EV yield calculated by particles per mass of lymph node in isolated PBS-, CFA + PBS-, and OVA + CFA immunized mice group. Variation of total protein content per total particles number in the lEV and sEV subpopulations of the PBS control group (**a**), CFA + PBS group (**b**), and OVA + CFA treated group (**c**). Additionally, the variation of total lipid content per total particle number in the lEV and sEV subpopulations of PBS- (**d**), CFA + PBS- (**e**), and OVA + CFA groups (**f**). The EV yield was calculated as particles per lymph node mass (g) in the PBS group (**g**), the CFA + PBS group (**h**), and the OVA + CFA group (**i**). Statistical significance was calculated by paired t-test, * $p < 0.05$; n = 8 (protein per particles), n = 6 (lipid per particles), n = 9 (EV yield).

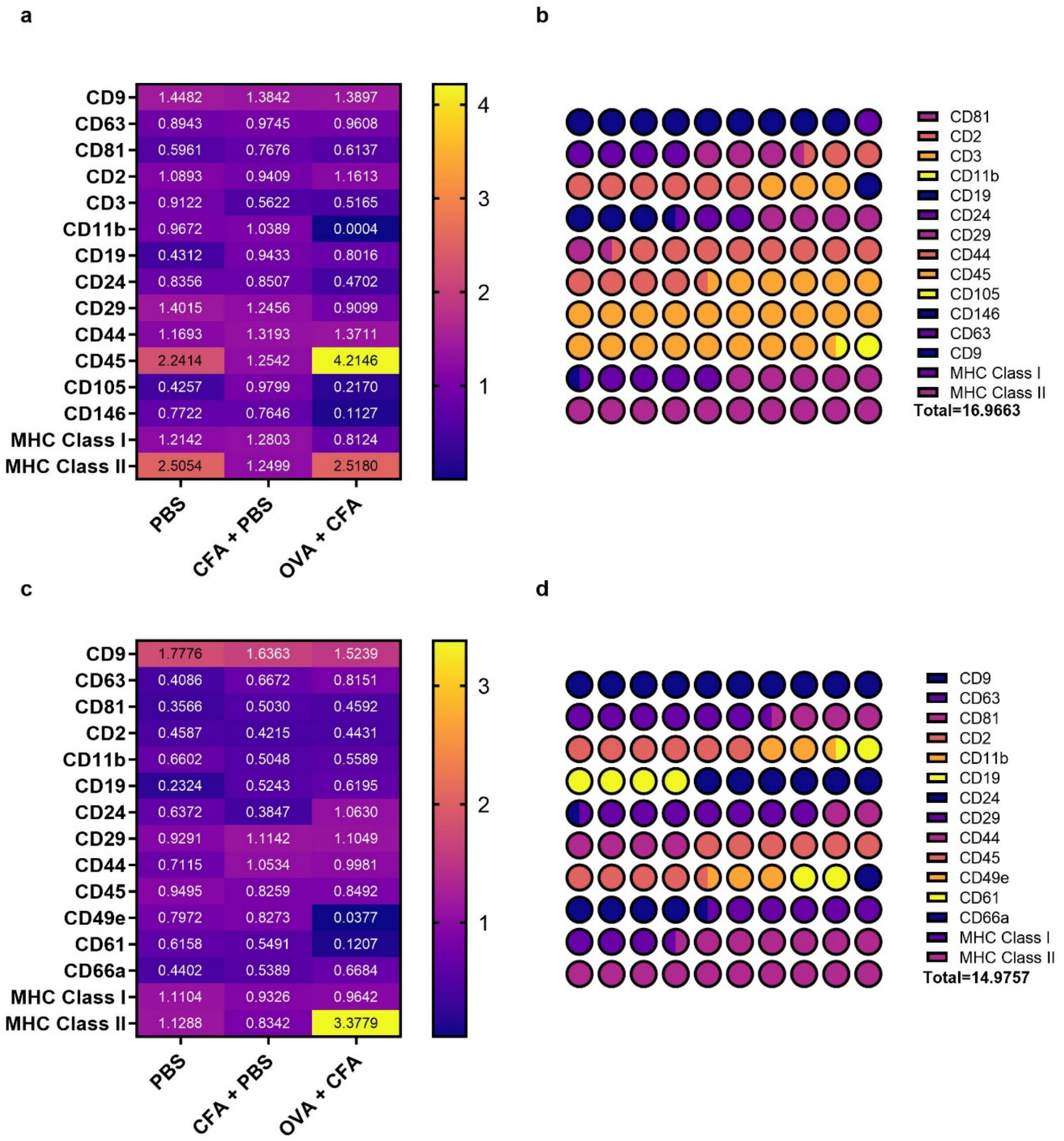

Supplementary Figure S6: MACSPlex APC bead-based EV kit for immuno-oncology for detection of potential surface markers of EV subtypes. Heatmap (**a**) graph represents the lEV subpopulation of all groups, and Parts of whole (**b**) represents the lEV subpopulation of OVA + CFA treated group, while the heatmap (**c**) present the sEV population of all groups and Parts of whole graph (**d**) shows the sEV subpopulation of OVA + CFA treated group. The figures depict only the levels of the most abundant 15 markers in the PBS control group, plus 2 isotype controls in PBS control -, CFA + PBS -, and OVA + CFA adjuvant group; n = 3.

a

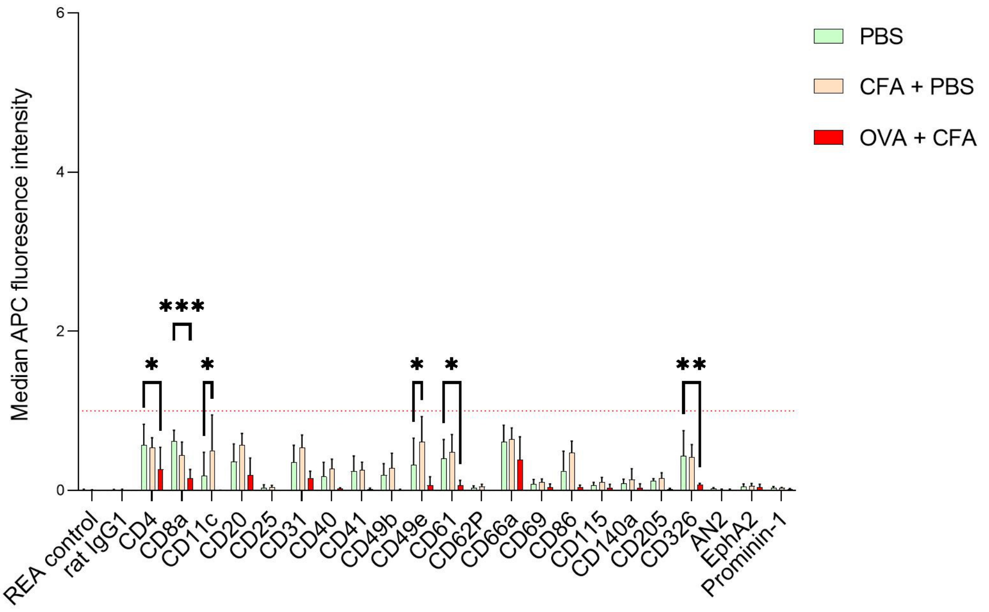

b

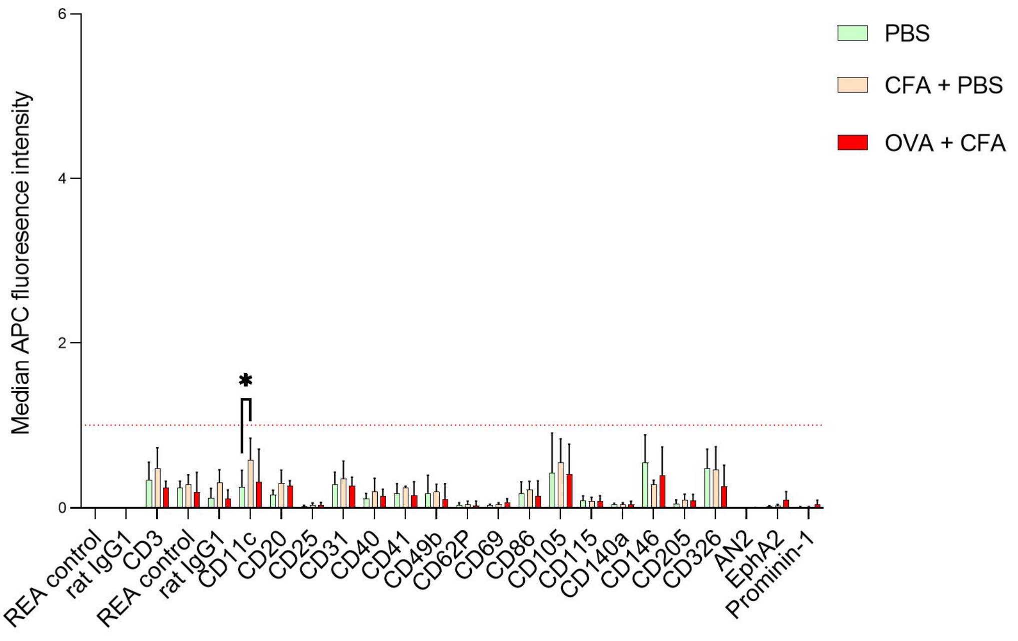

Supplementary Figure S7: MACSPlex bead-based analysis of two isotype controls plus remaining 22 markers that do not exhibit high prominence. Graphs illustrating the lower marker levels of the remaining 22 surface markers are represented in lEV (**a**) subpopulations and sEV

subpopulations (**b**) which are below fold change 1. Statistical significance was assessed using two-way ANOVA, * $p < 0.05$; ** $p < 0.01$, *** $p < 0.001$; n = 3.

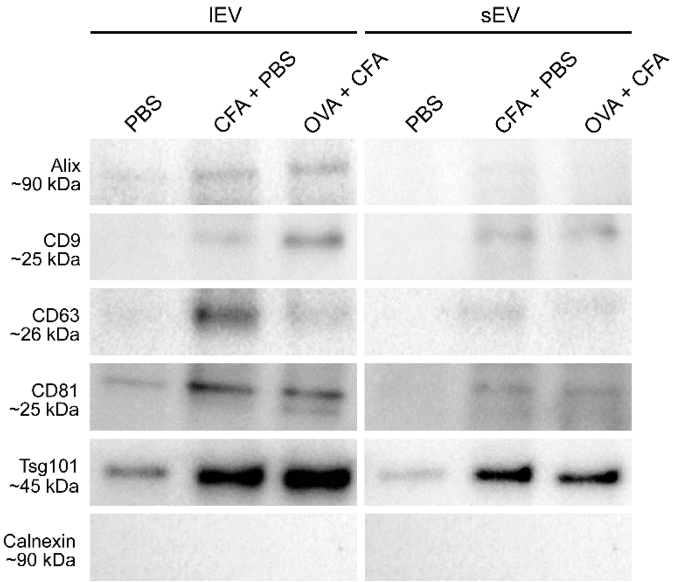

Supplementary Figure S8: Western blot protein analysis validated the presence of extracellular vesicle protein markers in the PBS-, CFA + PBS-, and OVA + CFA-injected groups of mice. The proteins investigated included Alix, CD9, CD63, CD81, TSG-101 which were detectable and showed increased expression in the CFA + PBS and OVA + CFA immunized groups. The anti-CD63 antibody detects the C-terminal of the CD63 marker protein, and the observed band is consistent with the validation data provided by the antibody supplier. A marker of endoplasmic reticulum, Calnexin, was also tested and found to be absent.

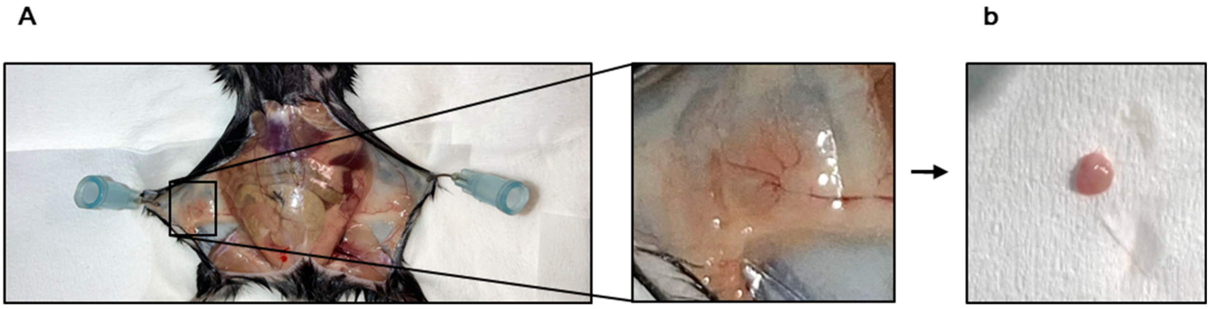

Supplementary Figure S9: This image illustrates the identification and removal of the iLNs from the mouse. The photo shows the location of the iLN in a mouse (**a**), as well as the iLN after its excision (**b**).

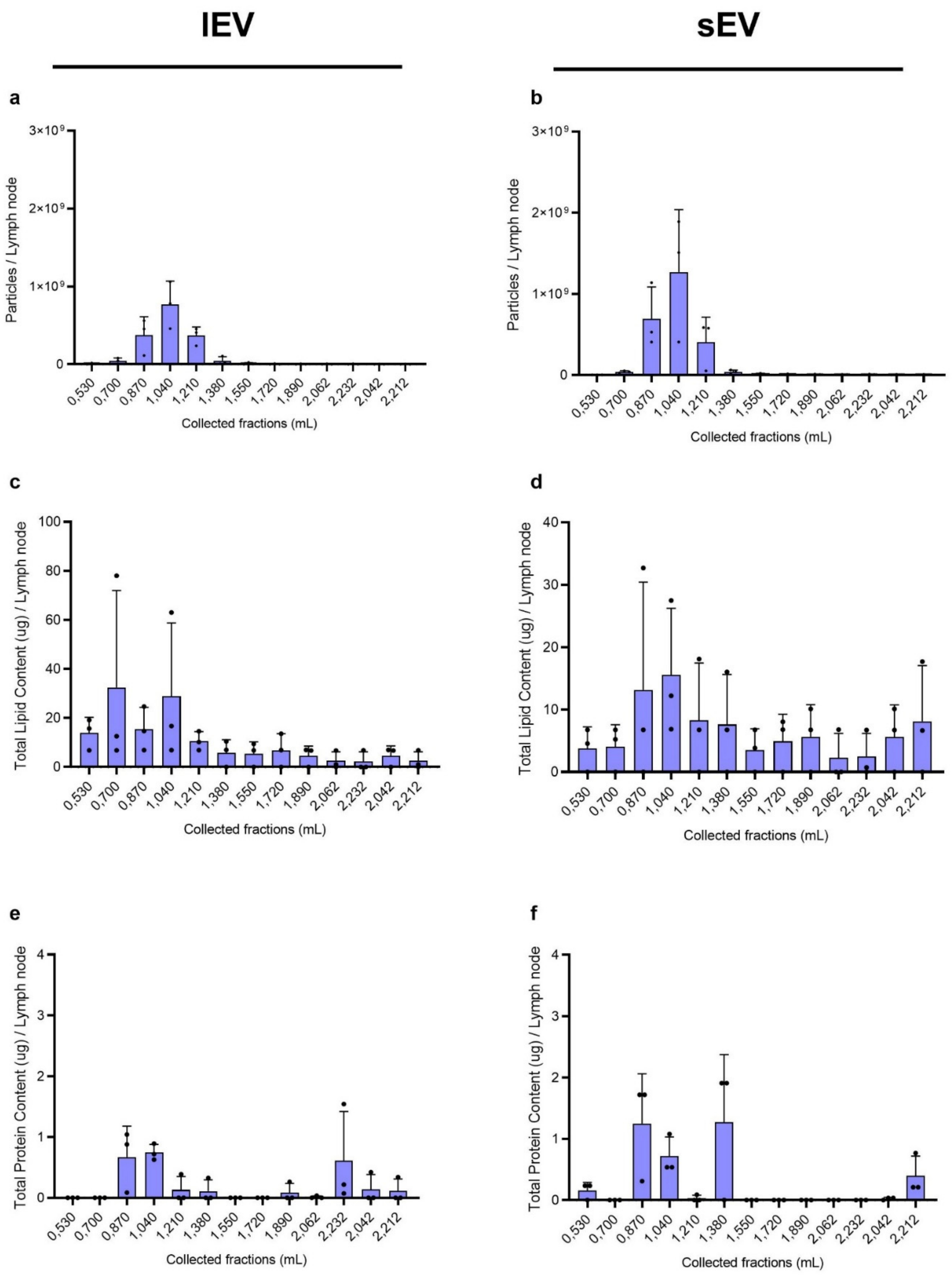

Supplementary Figure S10: Measurement of various parameters of different fractions collected by size-exclusion chromatography during EV isolation from differential centrifugation. Particle/ lymph node quantification of lEV and sEV subpopulations were

performed using Nanoparticle Tracking Analysis (**a**), while the Total Lipid Content was assessed by SPV Assay (**b**), and the Total Protein Content was determined by BCA Assay (**c**). The analysis demonstrated the potential fractions considered of containing EVs can be localized from 0.700 mL until the point of overall 1.380 mL of elution. n = 3.

a

**Dye**	Trypan Blue solution	eBioscienceTM Fixable Viability Dye
**Conjugate**	-	eFluorTM 780
**Color**	Dark blue	Infrared
**Excitation wavelength**	-	633 nm
**Emission wavelength**	-	780 nm
**Manufacturer**	Merck, Rahway, New Jersey, U.S.	Thermo Fisher Scientific, Waltham, Massachusetts, U.S.
**Catalog number**	T8154-20ML	65-0865

b

**Target**	CD16/32	CD3	CD4	CD8b
**Reactivity**	Mouse	Mouse	Mouse	Mouse
**Host species**	Rat	Rat	Rat	Rat
**Clonality**	Monoclonal	Monoclonal	Monoclonal	Monoclonal
**Clone number**	93	17A2	RM4-5	YTS156.7.7
**Conjugate**	Purified	PerCP/Cy5.5	Brilliant Violet 785™	FITC

**Dilution**	1.0 µg per million cells in 100 µl volume	1.0 µg per million cells in 100 µl volume	0.25 µg per million cells in 100 µl volume	0.25 µg per million cells in 100 µl volume
**Excitation wavelength**	-	482 nm	405 nm	495 nm
**Emission wavelength**	-	690 nm	785 nm	519 nm
**Manufacturer**	Sony Biotechnology, San Jose, California, U.S.	Sony Biotechnology, San Jose, California, U.S.	Sony Biotechnology, San Jose, California, U.S.	BioLegend, San Diego, California, U.S.
**Catalog number**	1106505	1101085	1102760	126605

c

	**Antibody**	**Target**	**Host**	**Clonality**	**Dilution**	**Condition**	**Catalog number**	**Company**
**Primary antibody**	Alix	human, mouse	mouse	Monoclonal	1:1000	Reducing	634502	BioLegend, San Diego, California, U.S.
	Calnexin	human, mouse, rat	rabbit	Polyclonal	1:1000	Reducing	ab22595	Abcam, Cambridge, UK
	CD9	mouse, rat, human	rabbit	Polyclonal	1:2000	Reducing	SAB4503606	Merck, Rahway, New Jersey, U.S.
	CD63 C-terminal	mouse	rabbit	Polyclonal	1:750	Non-reducing	SAB2109138	Merck, Rahway, New Jersey, U.S.
	CD81	rat, human, mouse	rabbit	Polyclonal	1:1000	Reducing	SAB3500454	Merck, Rahway, New Jersey, U.S.

	TSG-101	mouse, rat, human	rabbit	Polyclonal	1:1000	Reducing	HPA006161	Merck, Rahway, New Jersey, U.S.
**Secondary**	HRP Goat anti-mouse IgG	mouse	goat	Polyclonal	1:2000	-	405306	BioLegend, San Diego, California, U.S.
	HRP Goat anti-rabbit IgG	rabbit	goat	Polyclonal	1:2000	-	7074P2	Cell Signaling

Supplementary Table S1: A table represents the data of used viability dye and antibodies. Reagent dye for viability analysis of cells for cell counts and flow cytometer (**a**) and data of antibodies used for immunophenotyping of cells by flow cytometer (**b**). Primary and secondary antibody tests related to the Western blot experiment (**c**).
